# A Process Evaluation on the Implementation of Cross-Organizational Intergenerational Partnerships

**DOI:** 10.1093/geroni/igaa057.2606

**Published:** 2020-12-16

**Authors:** Carson De Fries, Andrew Steward, Rachel Fix, Leslie Hasche

**Affiliations:** 1 University of Denver, Denver, Colorado, United States; 2 University of Denver - Graduate School of Social Work, Denver, Colorado, United States

## Abstract

A collaborative group of organizations in the rocky mountain region of the U.S. implemented sixteen intergenerational programs with varying themes (i.e. mentorship, music and art therapy, baking, life stories, etc.). This two-year study used survey data and a focus group to understand successful approaches and challenges to implementing cross-organizational partnerships between adult-serving and youth-serving organizations. We utilized qualitative data through open-ended comments on program evaluations and a focus group discussion with leaders of seven community organizations. Questions in the focus group prompted participants to describe the process of initiating, developing, and sustaining partnerships. The three main themes were: the benefits of shared organizational values, outlining programmatic roles and expectations, and an ability to cope with negative partnership experiences. Insights about successful approaches to implementing cross-organizational partnerships, as well as the challenges of developing and sustaining such partnerships may be beneficial in similar settings.

